# The Play of Genes and Non-genetic Factors on Type 2 Diabetes

**DOI:** 10.3389/fpubh.2019.00349

**Published:** 2019-11-19

**Authors:** Michael Mambiya, Mengke Shang, Yue Wang, Qian Li, Shan Liu, Luping Yang, Qian Zhang, Kaili Zhang, Mengwei Liu, Fangfang Nie, Fanxin Zeng, Wanyang Liu

**Affiliations:** Department of Nutrition and Food Hygiene, School of Public Health, China Medical University, Shenyang, China

**Keywords:** non-genetic factors, gene, environmental factors, type 2 diabetes, variants

## Abstract

Diabetes has been a disease of public health concern for a number of decades. It was in the 1930s when scientists made an interesting discovery that the disease is actually divided into two types as some patients were insensitive to insulin treatment then. Type 2 Diabetes which happens to be the non-insulin dependent one is the most common form of the disease and is caused by the interaction between genetic and non-genetic factors. Despite conflicting results, numerous studies have identified genetic and non-genetic factors associated with this common type of diabetes. This review has summarized literature on some genes and non-genetic factors which have been identified to be associated with Type 2 diabetes. It has sourced literature from PubMed, Web of Science and Medline without any limitation to regions, publication types, or languages. The paper has started with the introduction, the play of non-genetic factors, the impact of genes in general, and ended with the interaction between some genes and environmental factors.

## Introduction

Diabetes has been a disease of public health concern for a number of decades. It was in the 1930s when scientists made an interesting discovery that the disease is actually divided into two types, as some patients were insensitive to insulin treatment then (1). Since then, the study of the disease took a new twist with researchers looking at it in different perspectives. Although all the types of diabetes result in high blood sugar levels over a prolonged period, Type 1 diabetes is said to be an autoimmune disorder which results in the destruction of insulin-producing pancreatic β-cells making it insulin dependent while Type 2 diabetes is non-insulin dependent (2). Another form reported in some papers was gestational diabetes happening in pregnant women due to the hormones produced during pregnancy (3). Type 2 diabetes, the most common type of this complex disorder, is said to account for 85% of cases (some studies put the figure closer to 90%) and generally happens at an older age of life (4).

WHO puts Diabetes as the seventh death causing disease, affecting most communities in the world. They projected that the number of people with diabetes may increase up to 522 million by 2030. This is in line with the dramatic increase in the disease prevalence from 108 million people affected in 1980 to about 422 million adults in 2014. The global prevalence (age-standardized) of the disease nearly doubled in the same period, increasing from 4.7 to 8.5% in the adult population (5). The primary cause of death in individuals with diabetes is not the disease itself but its complications. If compared to the general non-diabetic population, patients with Type 2 diabetes have about 7 years shorter life expectancy and this can be attributed directly to the effects brought by the major diabetic complications (6). The motility rate of people with Type 2 diabetes tend to increase with age with some studies suggesting that diabetic men exhibit a higher mortality risk than diabetic women (7, 8).

The study for the pathogenesis of Type 2 diabetes has taken so many interesting turns with time but as many chronic disorders, it is understood that the disease is as a result of some genetic predisposition and environmental factors. There are so many non-genetic factors that have been found and identified to be associated with Type 2 diabetes by different researchers in different studies. These factors range from lifestyle, food and its components to some toxins and pollutants (1, 9, 10). Although environmental factors may play a role in the development of Type 2 diabetes (11), even with the same environmental exposure, some individuals may be highly affected and become more susceptible to this complex disorder than others, confirming that heredity has its own impact on the disease. It was until the 1980s when genetic research generally advanced and made it possible to investigate and identify some loci which could explain the hereditary components.

This review therefore aims at bringing literature from different researchers on genetic and non-genetic factors that are associated with Type 2 Diabetes. This information may be used to help in sharpening up the knowledge of future researchers and also improve the understanding of different individuals on the prevention, progression as well as treatment of the disease.

## The Role of Non-Genetic Factors on Type 2 Diabetes

There are a number of non-genetic factors mentioned in different studies that are associated with Type 2 diabetes. The factors range from life style, food and its components, persistent organic pollutants as well as the gut ecosystem. Some of these factors also lead directly or indirectly to obesity and oxidative stress which are also known to be directly linked to Type 2 diabetes (12).

### Life Style Factors

One's way of living has an impact on their health and well-being. Dating back to two decades ago, a number of behaviors have been associated with many chronic disorders including diabetes (13). Despite having a few conflicting results, behaviors such as alcohol intake, smoking, exercises and sedentariness have been known to be associated with Type 2 diabetes for decades (14), however some more factors such as stress, insomnia (15) and the use of certain clinical drugs such as antibiotics (16) have emerged and found to be associated with the diseases in recent years. Stress has an effect on glucose metabolism through the activities of hypothalamic-pituitary-adrenal (HPA) axis as its chronic activation and joint mechanisms affect insulin activities and may cause insulin resistance and β-cell dysfunction (17). Although other papers suggest that the association between insomnia and Type 2 diabetes is mainly confounded by other factors such as alcohol intake, smoking, sex and stress (18), insomnia stimulates appetite regulating hormones causing unnecessary gaining of weight which causally result in obesity and is also associated with increased blood pressure and sympathetic nervous system activities which are all linked to insulin resistance and Type 2 diabetes (19). Not excluding other types of antibiotics, the exposure to Fluoroquinolone antibiotics which are used to treat variety of illnesses such as respiratory, urinary tract and other infections are said to be associated with insulin resistance as they cause oxidative stress (20) and have the capacity to penetrate cell membranes and induce an intracellular magnesium deficiency which are all linked to Type 2 diabetes (21).

### Food and Its Components

For decades nutrition and diets have been considered to play an important role in the development of many chronic disorders and the consumption of diets rich in certain food components such as saturated fats and high consumption of fast foods have all been linked to Type 2 diabetes (22). In recent years researchers have gone deeper to find out some more food components whose highly intake or deficiency can lead to Type 2 diabetes. It is necessary to investigate majority of micro nutrients as many prospective observational studies and clinical trials have shown that eating foods rich in or adequate intake or supplementation of some micronutrients or minerals such as Vitamin K (23), Vitamin D (24), Magnesium (25), and antioxidants such as β-carotene and α-tocopherol (26) has a reduced risk factor on Type 2 diabetes. Although other studies suggest otherwise, Vitamin K2 supplementation have shown that it could increase insulin sensitivity whereas Vitamin D plays a major role in insulin synthesis, release and sensitivity (23, 24, 27). Other vitamins such as Vitamin A or Retinol, Vitamins B, Vitamin C, or Ascorbic Acid and Vitamin E as well as their supplements have also been investigated to be associated with the disease but there results are conflicting (28). And what interests us is that there is a new evidence from a meta-regression analysis of some prospective cohort studies recently show that dietary magnesium intake is related to a reduced risk of Type 2 diabetes. A 100 mg/day increment in dietary magnesium intake was associated with an 8–13% reduction in risk of Type 2 diabetes (29).

Otherwise, studies in past two decades have also repeatedly mentioned that oxidative stress and inflammation are associated with Type 2 diabetes and this is mainly because of insufficient antioxidant defense. Evidence has it that low dietary intake of antioxidants such as β-carotene, α-tocopherol, and vitamin E play independent roles in the pathogenesis of Type 2 diabetes by increasing insulin resistance or impairing insulin action. Intake of diets with sufficient antioxidants, commonly fruits and vegetables, ensure a balanced defense mechanism on harmful effects of reactive oxygen species as they could prevent the gradual impairing of pancreatic β-cell, therefore reducing the risk of Type 2 diabetes (30, 31).

### Dietary Structures

Dietary patterns, including traditional, healthy, Mediterranean, and Western dietary patterns, have recently gotten great attention in the evaluation of the relationship between diet and health (32). And dietary patterns may influence health more than certain nutrients or nutritional groups and the identification of one desirable dietary structure has gotten a lot of attention in public health in recent years to prevent Type 2 diabetes (33).

The types and quality of diet compounds might be related with the risk of Type 2 diabetes, disease progress, and its secondary disease. Some researches carried out in the Western societies shown that Western dietary structure including higher intake of red meat, processed meat, and refined grains was significantly associated with increased risk of Type 2 diabetes (34). Meanwhile, Erber et al. also found that an increased risk of diabetes was observed for participants adhering to a Western diet (35). One research from Greenland showed that the traditional food (mostly consisting of marine mammals and fish) was positively associated with Type 2 diabetes, impaired fasting glucose (IFG), and fasting plasma glucose (36). And a latest systematic review from de Carvalho et al. presented that Vegetarian, vegan, Mediterranean, and Dietary Approaches to Stop Hypertension dietary patterns reduced 0.8% on average of percentage of glycated hemoglobin, meanwhile, the reduction in fasting glycemia and improvement in Homeostasis Model Assessment of insulin sensitivity was also observed, considering all included studies (37). In addition, another umbrella review of meta-analyses of prospective observational studies found that dietary factors have an critical role in the primary prevention of Type 2 diabetes, and the study also pointed out that the high quality of evidence was only targeted at associations for incidence of Type 2 diabetes with moderate alcohol consumption, cereal fiber, whole grains, red meat, processed meat, bacon, and sugar sweetened beverages (38).

### Role of Socioeconomic Status on Type 2 Diabetes

The public health of population groups links with socioeconomic status (SES) inextricably in different ways. SES is composed of many dimensions and is often measured by several index, such as income, education and occupation. SES has been recognized as an important determinate factor of population's health in both industrialized and developing countries (39). And the association between SES and Type 2 diabetes, which depends on the stage of economic and social development, differs worldwide (40, 41). The relationship between SES and Type 2 diabetes was positive several decades ago but is now negative in developed countries while in developing societies the relationship remains positive (41–43). What's more, a systematic review from Wu et al. found that low education is probably associated with an increased prevalence of Type 2 diabetes in China (40). And education in men and the measure of income in both genders is positively related to self-reported and total diabetes prevalence and awareness of diabetes in a Chinese population aged 45 years or older (44). Also, a latest finding from Tang et al. found economic development, income and gender differences also played a critical role in Type 2 diabetes (45).

### Regional Imbalance on Type 2 Diabetes

Practically, diversity in natural environments, ethnic differences and social environment differences such as economic status, dietary structure, lifestyle, physical activity et al. in different regions, all of them might have impacts on Type 2 diabetes partly, and thus the integrated effects might result in regional imbalance on Type 2 diabetes. For example, different studies have observed different effects of fish intake on the risk of Type 2 diabetes (46). In reality, these differences can be put down to the geographical diversity of the studied regions. Fish intake in Europe and North America was within recommended intake, while in Asian countries, it was correlated with the decreased risk of Type 2 diabetes (46). Although the reason has not been recognized yet, it might be due to the differences in kinds of the consumed fish, type of cooking as well as the differences in the types and level of exposure to some levels of some contaminants (46). For the same ethnic population in societies at different levels of development may be observed for Chinese communities in Hong Kong, where there is a negative association between socioeconomic status and Type 2 diabetes, and Mainland China, where there is a positive association (47). Those research results might remind us that different and diverse factors existing in different regions might affect the incidence and prevalence of Type 2 diabetes which in return result in regional imbalance of Type 2 diabetes.

### Persistent Organic Pollutants (POPs)

POPs which are mainly classified into five main types (polychloro-dibenzopara dioxins (PCDD), polychloro-dibenzo furans (PCBF), poly-chlorinated biphenyls (PCBs), organo-chlorine (OC) pesticides, and poly-brominated flame retardants) are said to be organic compounds that are/were produced as industrial chemicals or byproducts and have a resistant characteristic to any form of degradation process and find their way into the food web, resulting in potential effects on human health by accumulating in lipid containing tissues such as adipose tissue and circulating in the body while bound to lipids (48). polychloro-dibenzopara dioxins (PCDD), polychloro-dibenzo furans (PCBF), poly-chlorinated biphenyls (PCBs), organo-chlorine (OC) pesticides are Organo-chlorine compounds whose production and use were banned in the 1970s because of their toxicity characteristics but their residues are still found in water and soil and humans' exposure to Organo-chlorine compounds and POPs in general is mainly through the intake of animal fats by the consumption of meat, fish and daily products (49). Activities of a number of active low materials found in POPs such as γ-aminobutyric acid disturb the ion channel systems that are beneficial for regular pancreatic function thereby causing glucose homeostasis (50).

A number of individual POPs have been investigated through epidemiological and laboratory based studies for over two decades now. Epidemiological studies and animal studies which started a decade ago indicated that exposure to some organochlorine pesticides (OC) is associated with obesity, dyslipidemia, and insulin resistance (51–53). A recent study done in Taiwan found that exposure to high dioxin level (PCDD/Fs ≥20 pg WHO98-TEQDF/g lipid), a byproduct of herbicide production and paper bleaching increase the risk to Type 2 diabetes (54). These findings were in line with another study done in Germany where the risk of Type 2 diabetes increased in workers exposed to 2,3,7,8-tetrachlorodibenzo-*p*-dioxin (TCDD) in a phenoxy herbicides factory whereas that differed from one study done in the USA where the re-analysis of a study on military workers during the Vietnam war showed that there was no risk after exposure to Orange and its contaminant TCDD (55). Exposure to another compound Bisphenol A (BPA) which is the starting material for the production of plastics has an effect on insulin synthesis, secretion in pancreatic beta cells and modifies insulin signal in the liver, skeletal muscle and adipose tissue, all of which lead to insulin resistance, obesity and Type 2 diabetes (56). A recent study concluded that elevated POP exposure has diabetogenic potential after investigating 3 organochlorine pesticides [hexachlorobenzene (HCB, β-hexachlorocyclohexane (β-HCH) and p,p'-dichlorodiphenyldichloroethylene (p,p'-DDE)] and measuring 20 polychlorinated biphenyls (PCBs) in blanked plasma (57). This corroborated most recent review on POPs that exposure to Dichlorodiphenyl dichloroethylene (DDE), a major metabolite of dichlorodiphenyltrichloroethane (DDT) and trans-nonachlor, were confirmed to be associated with the disease (58).

### Role of the Gut Ecosystem on Type 2 Diabetes

The collection of all microorganism resident in the gastrointestinal tract is referred to as gut microbiota. The human gastrointestinal tract is residence to an estimated 100 trillion bacteria, almost 10 times the total number of cells in the human body (59). Firmicutes, Bacteroidetes, Actinobacteria, and Proteobacteria are the four major phyla in the gut ecosystem. On top of playing a vital role in the digestion process, the gut microbiota is vital in the maintenance of one's individual health, but is also reported to be associated with the pathogenesis of various metabolic diseases including Obesity and Type 2 Diabetes (60, 61). Results in a number of recent studies have suggest that gut microbiota in individuals with Type 2 diabetes differs from that of non-diabetic individuals (62), such that there is a co relationship that exists between Type 2 diabetes and increased abundance of Lactobacillus, lowered abundance of butyrate-producing microbes and fewer Clostridia (and more Firmicutes), suggesting that that Proteobacteria and Gram-negative Bacteroidetes activities do induce the pathogenesis of Type 2 diabetes (62, 63). Scientists have gone on to say that these microbiome can exist as new biomarker for Type 2 diabetes prediction (64). The is also a relation present between diet and gut microbiota, that alters the autoimmunity and energy balance, causing inflammation and metabolic dysfunction including Type 2 diabetes (65).

## The Role of Genetics on Type 2 Diabetes

Although the non-genetic factors play a major role in the development of Type 2 diabetes, even with the same exposure, the susceptibility to the disease will vary between individuals which make it possible for researchers to conclude that the disease is as a result of heredity as well. Heredity has all along been mentioned to play an important role in the development of diabetes but it was until 3 decades ago (in the 1980s) that genetic variants started to be identified to back up these heredity claims after the advancement of human genetic studies and the coming in of modern genetic technologies (10, 66). Candidate gene studies were the ancient studies used in identifying genes that are associated with Type 2 diabetes before the coming of genome wide linkage and genome wide association studies but only a few genes and variants were identified (67).

### Candidate Gene Studies on Type 2 Diabetes

Candidate gene study approach, focusing on genes that are in some way related to the disease or with prior knowledge to the gene function, were the ancient research studies used in identifying genes associated with chronic diseases including Type 2 diabetes. By the year 2000 Candidate gene studies had identified a number of genes to be associated with Type 2 diabetes although the approach was being criticized for lacking roughness and sensitivity as some genes which were already known to have an impact on insulin activities were not confirmed in population studies as they showed association and having only investigated a limited number of variants and genes in limited, small or unmatched populations that made it difficult to replicate weak associations that were detected (68, 69). This prompted researchers to come up with large scale candidate genes association studies. One research group in the United Kingdom investigated 152 single polymorphisms in 71 candidate genes in 2003 and found *ABCC8* (sulphonylurea receptor), *KCNJ11* (KIR6.2), *SLC2A2* (GLUT2), *HNF4A* (HNF4α), and *INS* genes having an effect on pancreatic β-cell function there by inducing insulin resistance and another three genes, *INSR, PIK3R1*, and *SOS1* to be associated with insulin action. Replication studies confirmed these results (70). A similar large scale study was also performed in Japan (71) and was followed by another one in Germany 5 years later where some more genes (*SLC27A5, FABP6, Thr79Met, DBI, PTGES2*, and *CLPS*) were identified (72). Genes such as *RAPGEF1*, HNF1 homeobox A *(HNF1A)* and HNF1 homeoboxB *(HNF1B)* were also identified with the same approach.

### Genetic Linkage Studies on Type 2 Diabetes

Linkage is a genetic tendency where genetic markers are inherited together as a result of being near to one another on the same chromosome. Genetic linkage analysis, one of the old study approaches, focuses on genomic regions with large genetic effect that can influence the development of a disease (73, 74). Genome-wide linkage scans have proved that some genes are associated with Type 2 diabetes on specific chromosomes such as 4q, 12q, and 22q in AA, 6p in EA, 2p in AI and 13q in African American (75), 3p at marker D3S2406 in Mexicans Americans (76) and 2p and 13p of Chinese (77) increase the risk of developing the disease. Linkage studies have been used to identify genes associated with different chronic disorders with unknown pathogenesis such as Type 2 diabetes, but they are considered to be unsuccessful by many researchers because they have only identified a few genes (75). Many studies, including one review (published in 2013) reported that these studies were only successful in reviewing two genes [calpain-10*CAPN10* and transcription factor 7-like 2 (T-cell specific, HMG-box) (*TCF7L2*)] (10) associated with Type 2 diabetes although this differs from what was reported earlier where three more genes *HNF4A, ENPP1, ADIPOQ* were included (78).

### Genome Wide Association Studies on Type 2 Diabetes

Until the early 2000s, so many candidate genes and linkage studies have been done but only succeeded in identifying very few susceptibility loci and the coming of genome wide association studies which focuses on searching single nucleotide polymorphisms (SNPs) that happen more frequent in people having a certain disease brought an interesting twist and is thought to be a breakthrough concerning the genetic research on Type 2 diabetes (10, 66, 79). Genes such *KLF14, ENPP1, ADAMTS9, ADIPOQ, IRS, GCKR, SREBF1, FTO HNF4A, NOTCH2, IGF2BP2, CDKAL1, JAZF1, SCL30A8, HHEX, TCF7L2, EXT2, FTO* and many others have been identified by this approach. By 2009 GWAS had identified 19 more SNPs associated with the Type 2 diabetes (80) and this number increased to more than 60 SNPs by 2016 in Asian and European ancestry alone with researchers not only identified the variants and the genes involved but also validated the effects conferred by these reported common variants in different populations (81) as conferred impacts may differ or might be the same but with substantial differences in allele frequencies as it is the evident with Chinese and European populations on variants in *IGF2BP2, CDKAL1, JAZF1, SCL30A8, HHEX, TCF7L2, EXT2*, and *FTO* (82) and Europeans and North African Arabs in variants in TCF7L2 and other genes (83) which made it possible to conclude that biological actions and activities of common variants may be similar in different ethnic groups.

The advancement in genetic tools and the coming of genome-wide association studies have also made it possible for researchers to do some comprehensive pharmacogenomics studies on variability in drug response and improve their understanding on why some patients develop drug resistance to Type 2 diabetes pharmacological treatment as the first recommended approach after failure in lifestyle changes. Researchers have identified more SNPs and genes involved in drug metabolism such as *CYP2C9* and those involved in insulin signaling such as *KCNJ11* and *PPARG* (84). GWAS have reviewed how certain variants and genes also have an impact on individual Type 2 diabetic drugs such as variants in *ABCC8* and *KCNJ11* on oldest oral agent Sulfonylureas as well as *SLC12A1* and AQP2 on Thiazolidinediones (TZDs),which all help clinicians in making direct individual decisions on efficient dosages for prevention, treatment and management of Type 2 diabetes (85).

## Gene-environmental Interaction and the Risk of Type 2 Diabetes

The interaction of genes and environment factors result in genotype plasticity and has become one of the interesting areas of study in finding the pathogenesis of different chronic diseases (86). The advancement in research tools on both genetic and environmental research has made it possible to identify variants and environmental factors that interact and increase the risk on Type 2 diabetes. In Chinese population, the interaction between an organochloride which is one of the isomers of hexachlorocyclohexane (HCH) called (β-hexachlorocyclohexane (β-HCH) and variants of *ADIPOQ* gene is said to be associated with Type 2 diabetes risk (52) where as *SLC30A8* rs13266634 which on its own is associated with higher odds of the Type 2 diabetes has a modification effect on plasma zinc concentration association with the disease, where its lower odds association with Type 2 diabetes is nullified (87). Recent studies in the USA have reported negative association between two paired genes and environmental factors interactions. One was between variant rs1092393 found in *NOTCH2* gene and heptachlor epoxide (a byproduct of insecticide Heptachlor) and another between the same variant and cis-b-carotene. Both the gene and its variants and the environmental factors mentioned here have been identified to be associated with Type 2 diabetes in isolation in different genetic and environmental studies recently (9). Dietary carbohydrates and fiber were also found to be modifiers of some genetic variants which were associated with Type 2 diabetes specifically for *ADAMT59* rs4607103, *DKN2A/2B* rs1801282, *FTO* rs8050136 in non-Hispanic whites, *ADAMT59* rs4607103, *THADA* rs7578597 in non-Hispanic blacks and *NOTCH2* rs1092398 and *TSPAN8-LGRS* rs7961581 in Mexican Americans (88).

### Epigenetics and Type 2 Diabetes

Despite advancement of genetic research tools, they have only been able to explain and identify genetic variants that increase the risk of Type 2 diabetes by 10.0–30.0% (89). Epigenetics is a field which can help in coming up of more understanding and clearance in the pathogenesis as it can provide a molecular link between genetics, environmental factors and Type 2 diabetes (90). Epigenetics which include DNA methylations, histone modifications, and microRNAs is the heritability alteration or change in gene functions, that do not involve changes in nucleotide sequence and is said to explain a portion of the pathogenesis of chronic diseases as it suggests that these modifications can be passed from one cell generation to the next and between generations of species as first evident in plant studies although there is minimal information of such in animal studies (91). In recent researches, a variety of epigenetic mechanisms and factors have been reported to increase the risk of developing Type 2 diabetes as they play vital roles in a number of cellular process. For example, maternal nutrition and environmental conditions such as famine result in the irreversible change in the methylation in infants and a research on mice showed that infants born from Gestational diabetes mellitus (GDM) mothers show hypermethylation and epigenetic down regulation of *IGF2* and *H19* genes which has an impact on insulin sensitivity (92). Epigenetic mechanisms also have an impact gene networks that are involved in insulin resistance and insufficiency as it controls genes like *GLP1 receptor* and *Paired box 4 (PAX4)* which are among the major genes that are associated with β-cell formation and functions (93). It is of much importance to conduct more human studies to explore more on the role of epigenetics on Type 2 diabetes and other chronic diseases as heritability that is observed and concluded may not be necessary as a result of inherited variations in DNA sequence, but a number of Epigenetic mechanisms and factors such as the intrauterine life observed in animal studies.

## Conclusion

Type 2 Diabetes Mellitus development is as a result of both environmental and genetic factors. This paper has outlined how both environmental and genetic factors contribute to the development of T2DM. Hope to provide some useful clues for in-depth studies regarding to study on the Type 2 diabetes in the future.

## Author Contributions

MM designed the paper and was responsible for the summarization of the review. MS contributed to the sourcing of data, selection of papers, data interpretation, manuscript development and revisions, approved the final version of the submitted manuscript, and was responsible for the summarization of the review. YW, QL, SL, LY, QZ, KZ, ML, FN, FZ, and WL contributed to data interpretation, manuscript development and revisions, and approved the final version of the submitted manuscript. WL was responsible for the supervision of the current work.

### Conflict of Interest

The authors declare that the research was conducted in the absence of any commercial or financial relationships that could be construed as a potential conflict of interest.
